# Response to: Importance of Comprehensive Reporting in Rare Pediatric Cases: Insights From a Cardiac Echinococcosis Case Report

**DOI:** 10.1177/21501351241292622

**Published:** 2024-11-06

**Authors:** Thierry Carrel

**Affiliations:** 30262 University Hospital Basel, Basel, Switzerland

**Keywords:** Echinococcosis, Cardiac resection, Pulmonary resection, Technique

We read with great interest the comment of Israr et al concerning our case report entitled “A Very Rare Cause of Dyspnea in a Child: A Hydatid Cyst from Echinococcus.”^1^ In this short report, we tried to present a pragmatic approach to a complex infectious problem in a pediatric patient. Israr and colleagues asked for more details regarding the patient's history, the diagnostic imaging, the operative technique and finally for some additional information regarding the follow-up.^2^

Israr and colleagues are absolutely right in saying that echinococcosis is an endemic disease in Usbekistan. The patient came from a small village far away from the capital, living in very simple conditions very close to domestic animals such as cows, goats, sheeps, camels, and dogs, just to enumerate the most common animals living among them. The hygienic situation in this village may have ranged from very simple to insufficient not only regarding the quality of the potable water but also for rinsing vegetable and fruits before eating. The past medical history of this child was unremarkable; he had no comorbidity at all and there was no other family member affected by echinococcosis.

For economic reasons (the patient's family has to pay the majority of the fees for medical services), therefore the diagnostic workup was kept very simple and included chest-x ray, echocardiography, and a computed tomography (CT)-scan. Of course, a minimal blood test analysis was performed and demonstrated significantly elevated sedimentation rate with a discrete leukocytosis with some degree of eosinophily. Serology was not performed because it would not have been necessary for proceeding with surgery.

Following removal of the pulmonary cyst, inspection of the intracardiac cyst was performed on the beating heart because the cyst was reached easily through the apex of the left ventricle. Since excision seemed to be a simple procedure due to the favorable location of the cyst, we did not expect any advantage of using cardioplegic arrest. The main benefits of this approach have been an expedient surgery with immediate return of normal contractility as soon as the left ventricle had been closed. We opted for this strategy due to our large experience with left ventricular assist device implantation on the beating heart. This technique is particularly easy when the aortic valve is competent. While doing so, special attention has to be directed at two points; the ventricle should never be completely empty and careful de-airing has to be performed through the ventriculotomy itself and through an aortic root vent.

Regarding the follow-up, the patient's condition was evaluated by a phone interview with the parents. This is how follow-up works for a large part of the population in the Usbek regions far away from the capital. We had information that the patient was doing clinically well, his weight was increasing very rapidly while the thorax deformity was smaller than what it had been before surgery. Other specific measures are unfortunately not possible in this patient population. The evaluation of the patient's (or family's) perspective around this type of surgery was unfortunately not possible not even through the local team. We agree with the authors of the letter that these points may add educational value for colleagues faced with similar cases.

## References

[1] Carrel T Sharipov I Vogt PR . A very rare cause of dyspnea in a child: a hydatid cyst from echinococcus. World J Pediatr Congenit Heart Surg. 2024;15(1):112-114. 37340730 10.1177/21501351231178757 PMC10729526

[2] Israr S Usama M Ikrama M . Importance of comprehensive reporting in rare pediatric cases: insights from a cardiac echinococcosis case report. World J Pediatr Congenit Heart Surg 2024 (in press). 10.1177/21501351241292619 39497479

